# Omphalolith presented with peritonitis: a case report

**DOI:** 10.4076/1757-1626-2-8191

**Published:** 2009-08-05

**Authors:** Hijran R Mahdi, Hany M El Hennawy

**Affiliations:** General Surgery Department, Al Khor Hospital, Hamad Medical CorporationQatar

## Abstract

Omphalolith is a hard, smooth, almost black bolus found in the umbilicus, resembling a malignant melanoma. It is often accompanied by seborrhea which may lead to abscess formation. It may be related to poor hygiene. Patient is usually complaining of umbilical discharge and pain. This report describes a rare case of omphalolith (umbilical stone) induced peritonitis, in a patient who presented as acute appendicitis. In our case the two umbilical stones found their way to the peritoneal cavity and induced peritonitis.

## Case presentation

**Figure 1.**
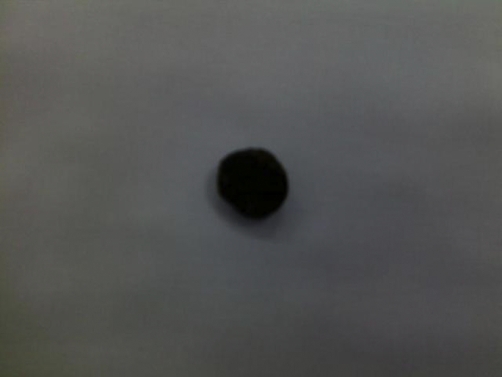
The umbilical stone.

**Figure 2.**
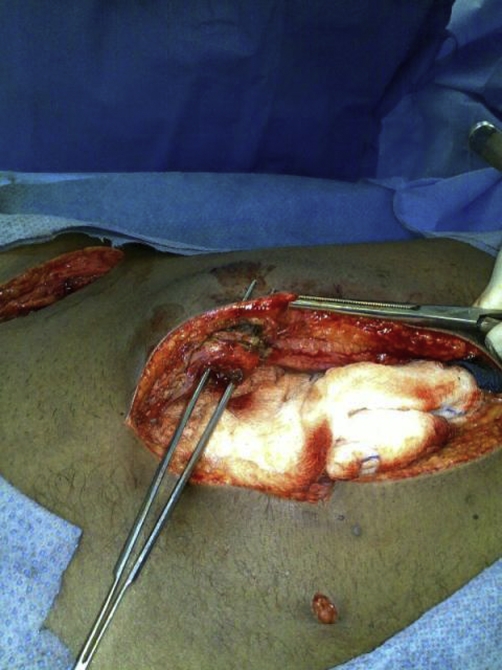
Showing the umbilical fistulous track which is probed by forceps.

**Figure 3.**
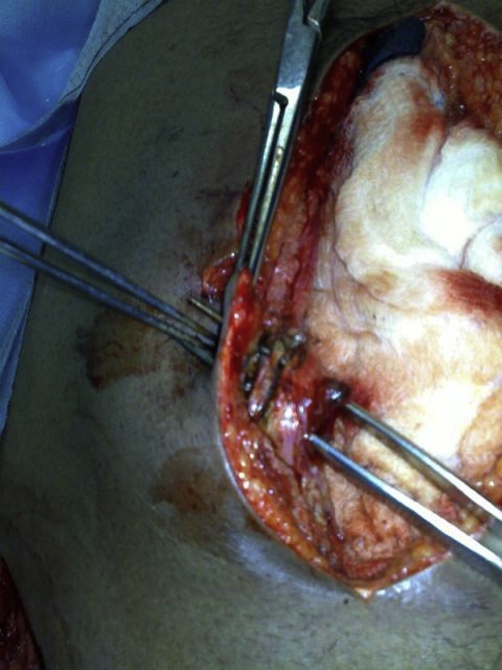
Showing the umbilical fistulous track which is probed by forceps.

**Figure 4.**
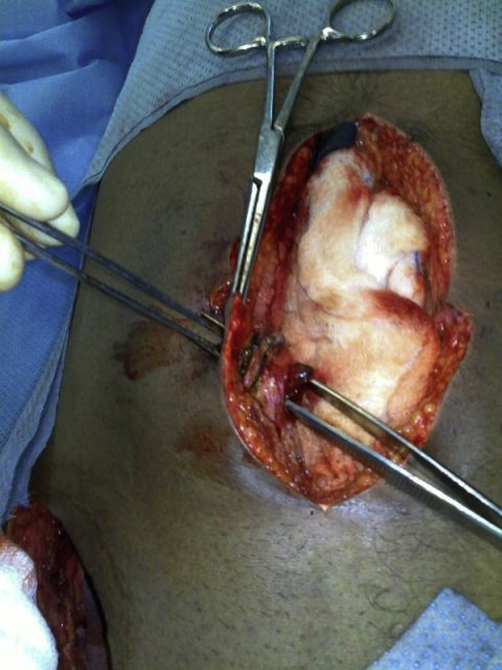
Showing the umbilical fistulous track which is probed by forceps.

We present a case of 26-year-old Nepalese male, laborer. He was admitted to our hospital from the Emergency Department complaining of right lower abdominal pain. The pain started seven days back with moderate severity dull aching pain started around umbilicus. The severity and frequency of the pain has increased during the last day. The pain is associated with clear malodorous mild umbilical discharge. This pain was none radiating, not related to meals, and not responded to over the counter analgesic, not associated with anorexia, nausea, vomiting, and fever or weight loss. No change in his bowel habits and no urinary symptoms. The patient has no history of surgeries, chronic illness or allergy and with no special habits. Physical examination was significant only for marked lower abdominal tenderness more on right iliac fossa with rebound tenderness in right iliac fossa and suprapubic region and positive cough test despite of analgesia and no obvious umbilical discharge or inflammation. Laboratory reports were within normal limits with white blood cell count of 14.4 thousand/ml. Chest X-ray: no air under the diaphragm. Initial clinical diagnosis was acute appendicitis. The patient was prepared and consented for open appendectomy. Grid iron incision was done. There was about 50-60 ml of yellowish thin odorless pus in the pelvis while the appendix looked normal. Appendectomy was done. Careful examination of terminal ileum revealed no Mickel’s diverticulitis. Decision was taken to do diagnostic laparoscopy; we introduced 10 ;mm trocar in the grid iron incision and closed the peritoneum around it using towel clips and sealed the whole area by opsite sheets. Careful inspection of lower abdomen and pelvis revealed no obvious abnormalities and as it was difficult to examine the duodenum from this approach we did insert another 10 ;mm port supraumbilical and one 5 ;mm port to the left of the umbilicus on mid clavicular line. Careful examination of gall bladder, liver, duodenum, stomach and spleen revealed no abnormalities. Again while examining the lower abdomen we found two firm brown fecolith between the bowel loops, here we thought that there was a colonic perforation somewhere. The fecolith were retrieved outside the abdomen (Figure 1). Careful examination of whole colon revealed no perforations, only area of induration over the transverse mesocolon. We used a hand assisted laparoscopy disc through the grid iron incision and again careful examination of whole small and large bowels revealed no abnormalities. Midline laparotomy incision was done and we did meticulous thorough examination of entire abdomen with the same result. Accidentally we noticed that the umbilicus looked abnormal with a perforation at its center and surrounded by area of severe induration. We probed the umbilical opening from inside outward and we found a well formed track (Figures 2, 3 and 4). Excision of the umbilicus including the fistulous track and some of the surrounding indurated tissues was done followed by Closure of midline and grid iron incisions. Histopathology examination revealed prominent acute and chronic inflammation suggestive of fistulous track with no granuloma or malignancy. The analysis of stones revealed keratin deposits. Post-operative period was smooth with good recovery. Patient was discharged home after 2 days.

## Discussion

Omphalolith is presented with a firm, black umbilical mass. It is easily removed with a warmed otic glycerin preparation. Histologic examination showed that it contained laminated keratin, amorphous material resembling sebum, numerous terminal hairs, and scattered collections of bacteria. Moderate amounts of argentaffin staining material were detected throughout the specimen, and the black color of the lesion was probably due to melanin and oxidized lipids, much like an open comedone. The mass is appropriately called an omphalokeratolith [1]. Differential diagnosis of the omphalic stone includes the so called umbilical cholesteatoma, an accumulation of crumbling, fetid masses in the umbilicus [2]. Omphalolith often accompanied by seborrhea which may lead to abscess formation [3]. Omphalolith is usually associated with bad hygiene and it could be detected by abdominal plain x-ray and abdominal CT scan. It can be removed by squeezing the stone from the umbilical foramen or through adequate opening of umbilical foramen under local anesthesia to the periumbilical area [4].
